# Treatment of Abscesses Connected with Caries of Bones

**Published:** 1851-06

**Authors:** 


*Treatment of Abscesses connected with Caries of Bone.*—The Surgical Society of Paris has been lately occupied with the discussion of a peculiar treatment of what the French surgeons call congestive abscess, excited by caries of bone. M. Boinet, who read a paper on the subject, proposes to evacuate the abscess as completely as possible, and to inject immediatly pure tincture of iodide. But when the sac is large, and for the first injection, he prefers adding equal parts of water to the tincture, and one drachm of iodide of potassium for every three ounces of the same. The addition of the salt is intended to facilitate the diffusion of the tincture. The author adds, that the general treatment should not be omitted, and advises the use of good diet, preparations of iron, iodide of iron, cod-liver oil, &c.

He brings forward four cases of cure where the abscess was situated in different parts of the body. The first case refers to a man, thirty-four years of age, who had been treated for five years for abscess connected with caries of the hip-joint; the latter affection was cured in eight months by M. Boinet's method. The second is that of a lady, thirty-eight years of age, who was cured of caries of the sacrum and abscess after eight injections. The third, a girl of twenty-one, suffering from caries of the third, fourth, and fifth cervical vertebræ; and the fourth, a child nine years of age, affected with caries and curvature of the spine, whose abscess contained more than a quart and a half of pus. The child was cured after five injections, and her health, which was much deteriorated, has improved remarkably since the operation. M. Boinet insists principally on the fact that his injections, besides modifying the walls of the sac, act directly upon the diseased bone, and that they prevent the absorption of the pus, whether the latter was putrid by the admission of air, or unaltered.

The Surgical Society discussed the subject with great animation; the majority of the members were very far from completely approving of the plan, and the general feeling was, that a great many more and varied trials must be made before this treatment can be entitled to any credit. It will certainly be worth while to give these injections a fair trial in this country; for the results of the usual treatment of caries and abscess are so uncertian, that it would be a great boon if M. Boinet's somewhat sanguine expectations became realized.—*Lon. Lancet.*

